# Supplementary material for: Patterns of Intron Gain and Loss in Fungi
Source: PLoS Biol. 2004 Nov 30;2(12):e422. doi: 10.1371/journal.pbio.0020422 (PMC532390; doi:10.1371/journal.pbio.0020422)
Supplement: Table S1 — Also available at http://genes.mit.edu/NielsenEtAl/. (4.3 MB ZIP). [file pbio.0020422.st001.zip › NielsenEtAl/html/513.html]

AN1056.1.NCU08485.1.MG06968.1.FG10870.1


```
 CLUSTAL W (1.82) Multiple Sequence Alignments - Introns Inserted


Sequence 1: NCU08485.1	396 aa
Sequence 2: MG06968.1	381 aa
Sequence 3: FG10870.1	393 aa
Sequence 4: AN1056.1	373 aa
Alignment Length: 404 aa
Number Identitical Residues: 111 aa
Alignment Score (without introns) 7222


MG06968.1 	------------MS2AQQAKRERRDAKSSNKYGKPIVLKSKILSAIADPRSSSSAILVAE
NCU08485.1	-MQSSDPRHFFETD2AEIAKRERREAKTKNTYGNPIKLKSKILGAVVDPRAPSSAIFVAE
FG10870.1 	-MQSADRDHFFETD2AVQAQRQRRAAKADNKNGEPIVMKSKVLAVVPDPAYPLTSVFIAE
AN1056.1  	MAVSSHRDDFFQTT2SALDEQRRKEAKSINTNGSPIRLQSKILAVKPDP-FDLGSVFVAQ
          	   ::      .   :   ::.*: **: *. *.** ::**:*..  **     ::::*:

MG06968.1 	SAGSVRHVKFG0DEPDSKTVYRGPSAPVTCVAVGGPNQKILFAGSWDKCIWSWNIDSQKP
NCU08485.1	SAGSVRRVQVD~GEGDTKTVYRGPTTPVSCVAVGGPGNKTVFAGAWDKNIWSWDIDTKAP
FG10870.1 	SAGAVRRINLE0TS-ELKTTYRGPKAPVTCVSIGGKDNQTVFAGSWDKDIWSWDVETGKS
AN1056.1  	SGGTVRKIVLA0TG-ETASIFKGPTAPITSLCFS-PNGRILFAGCWDKTIWSWDVNSTQR
          	*.*:**:: .     :  : ::**.:*::.:...  . : :***.*** ****::::   

MG06968.1 	ARKYAGHTDFIKTIVTARLGGTDILISGGADKRIIVWDVSTGKELHVLQDSVLNMLAVQA
NCU08485.1	GRKYAGHSDFVKAIVCARLGGKDVLVSGGADKKIIVWDIATGSKLHVLQDSVVNMLSIQD
FG10870.1 	GRKFSGHLDFIKTVVCATISGKHILISGGADKKIFVWDVESGKRLHTLQDPTTTMLAVQH
AN1056.1  	KLQYEGHTDFVRSVVTTRVNGQDLLVSGGADAQILVFDISSGQRLHVWKG---HAKGVQD
          	  :: ** **::::* : :.* .:*:***** :*:*:*: :*..**. :.      .:* 

MG06968.1 	LAIDPIQTT--VDEVYLFSASSDPHIRRWKIRLDSWKQVVDEVPQS-STERRTLLEHETS
NCU08485.1	LVVDPASSS--PDELCIISASSDPHIRRWKIRLNGWEQFAEPDPATPAAERHTILEHETT
FG10870.1 	IAIDPVLSD--STKLVFASASSDPHIRRWKITLDSYEQLPESFSDRPDAERLTIEEHETS
AN1056.1  	LLIDPLTLDDNSTEQIVFSAGSDREIRQFDLSKGSQDLTG----------TEALLAHDTN
          	: :**    ..  :  . **.** .**::.:  .. .               ::  *:*.

MG06968.1 	IYKLVFDQESDEVDLWTASGDGTAKCLARGSGFVSDDVFEHGDHVKAIAVTGT--WVITA
NCU08485.1	VYKLVIDHDGDEADLWSASGDGSAKCLSRIKGFTADDTFEHGDHVRAVAVTDQ--WVITA
FG10870.1 	IYRLFYDQSSEDVDLWTASADGSAKCLARSRNFVADDSYEHGDYVRGVLATEQ--WVITA
AN1056.1  	VYKLHFDQDG---DLWTASADKTTKCLIREDGWKANLTLTHPDYVKDVVVDERGGWVITA
          	:*:*  *:..   ***:**.* ::*** *  .: ::    * *:*: : .   ..*****

MG06968.1 	GRDEDIKFWDRS~TGTSYCSMQGHYDEVTDMVIVG------DSLCSVGIDGTIRTWPLTK
NCU08485.1	GRDEDLKVWDRT~SGKLYCSLEGHYDEVTELVILRGSVGSSERVCSVSIDGTIRTWPLDK
FG10870.1 	GRNEDVKIWDRS~SGKLYCTLIGHYEEVTDIVMLQDSAGTPRKVCSVGIDGTIRTWPLGK
AN1056.1  	CRDEEIRVWNRS0TGQLYHTFSGHYEEVTGLVLLG------TMLVSVSIDATIRQWSLKP
          	 *:*:::.*:*: :*  * :: ***:*** :*::         : **.**.*** *.*  

MG06968.1 	ALLDVAIKEQDADTEEKGKETGKEIGQEGLLSREEEAELAALMEDDD
NCU08485.1	ASLDAAVEEQ--KKPIKPAEEEKPDG-EGLLTADEEAELAALMEDDD
FG10870.1 	SELDEVVVKI--QKAAVPKEEEEENN-GNLLTAEEEAELAELMDD--
AN1056.1  	DDLRNAVAQA----TEHPTDEPSTNP-ESMLTEEEERELAELLEDD-
          	  *  .: :          :  .     .:*: :** *** *::*.
```
